# A Case of Left-Sided Infective Endocarditis of Multiple Native Valves Complicated by Valve Leaflet Perforation, Multiple Septic Emboli, Thromboembolic Events, and Cardiac Arrhythmias

**DOI:** 10.7759/cureus.31434

**Published:** 2022-11-13

**Authors:** Taqi A Rizvi, Waleed Sadiq, Shahkar Khan, Saud Bin Abdul Sattar, Sudeep Acharya, Michel Chalhoub

**Affiliations:** 1 Internal Medicine, Staten Island University Hospital, New York, USA; 2 Pulmonary and Critical Care Medicine, Staten Island University Hospital, New York, USA

**Keywords:** infective endocarditis, new onset afib, splenic infarcts, brain infarct, brain, septic emboli, acute pulmonary embolism

## Abstract

Coagulase-negative staphylococci (CoNS) can uncommonly cause native valve endocarditis. We present a case of left-sided infective endocarditis of native valves presenting with splenic, lung, and brain infarcts along with aortic and significant mitral valve involvement with mitral valve perforation. The patient was also found to be in atrial flutter and atrial fibrillation. Left-sided endocarditis is reported to cause brain and spleen infarcts but pulmonary embolisms are usually a complication of right-sided endocarditis. Atrial fibrillation is also known to increase mortality in patients with infective endocarditis.

## Introduction

*Staphylococcus epidermidis* is traditionally known to cause infections in implanted medical devices and is commonly found on the skin and mucous membranes [1,2]. However, coagulase-negative staphylococci (CoNS) causing native valve endocarditis (NVE) is still an uncommon occurrence. Infective endocarditis (IE) due to CoNS is associated with poor outcomes [2]. We present a case of a patient with left-sided IE presenting with deep vein thrombosis, pulmonary embolism, splenic, and brain infarcts along with aortic and significant mitral valve involvement with mitral valve perforation.

## Case presentation

A 44-year-old man with a past medical history of hypertension (not on any medication) and bilateral inguinal hernias presented to the hospital for palpitations and night sweats for the past two weeks. The patient started to feel unwell a few months before his presentation and was experiencing intense night sweats. He did not seek medical attention at that time and three months later started to notice dyspnea on exertion. He then progressively became more fatigued, lost interest in doing activities, and started experiencing what he referred to as panic attacks. He also noticed worsening lower extremity edema and weight gain. He was seen by his primary care provider who sent blood work and noticed elevated levels of adrenocorticotropic hormone, cortisol, and aldosterone and he was further referred to an endocrinologist. While being evaluated by the endocrinologist he heard a murmur noted on his physical exam and he was referred to cardiology who sent him to the emergency department due to tachycardia.

**Figure 1:**
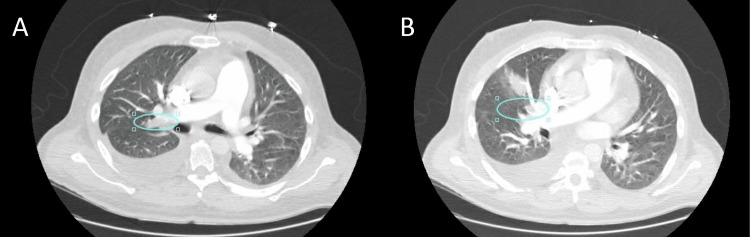
Multiple right-sided pulmonary emboli in (A) and (B).

The patient at the emergency department was hemodynamically stable but an electrocardiogram revealed atrial flutter with a rapid ventricular rate. The patient was further evaluated for suspected pulmonary embolism and imaging revealed right-sided pulmonary embolism and left deep vein thrombosis and he was subsequently admitted to the intensive care unit (Figure 1).

**Figure 2:**
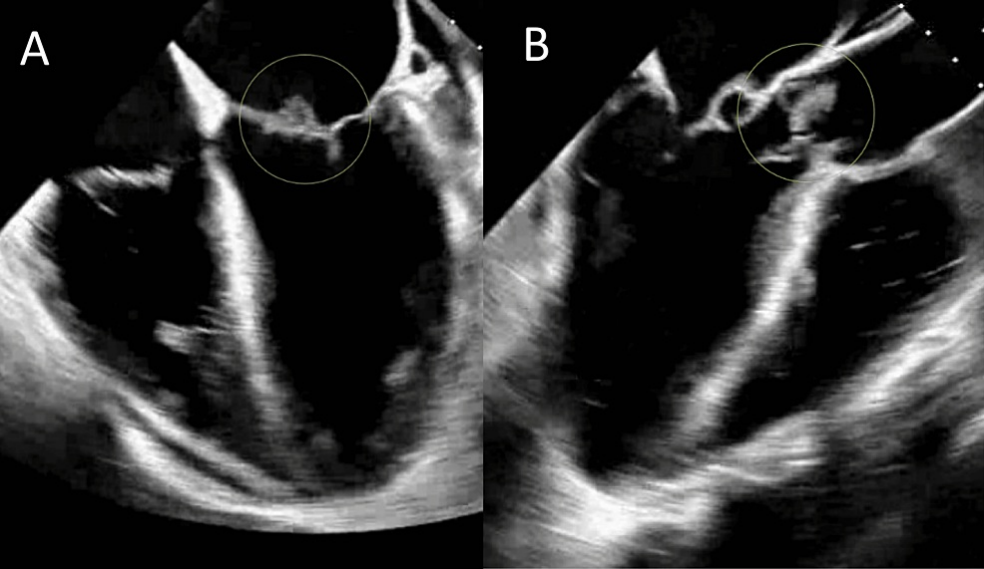
Vegetations of the mitral valve in picture (A) and aortic valve in picture (B) seen on the echocardiogram shown by circles around the vegetations.

**Figure 3:**
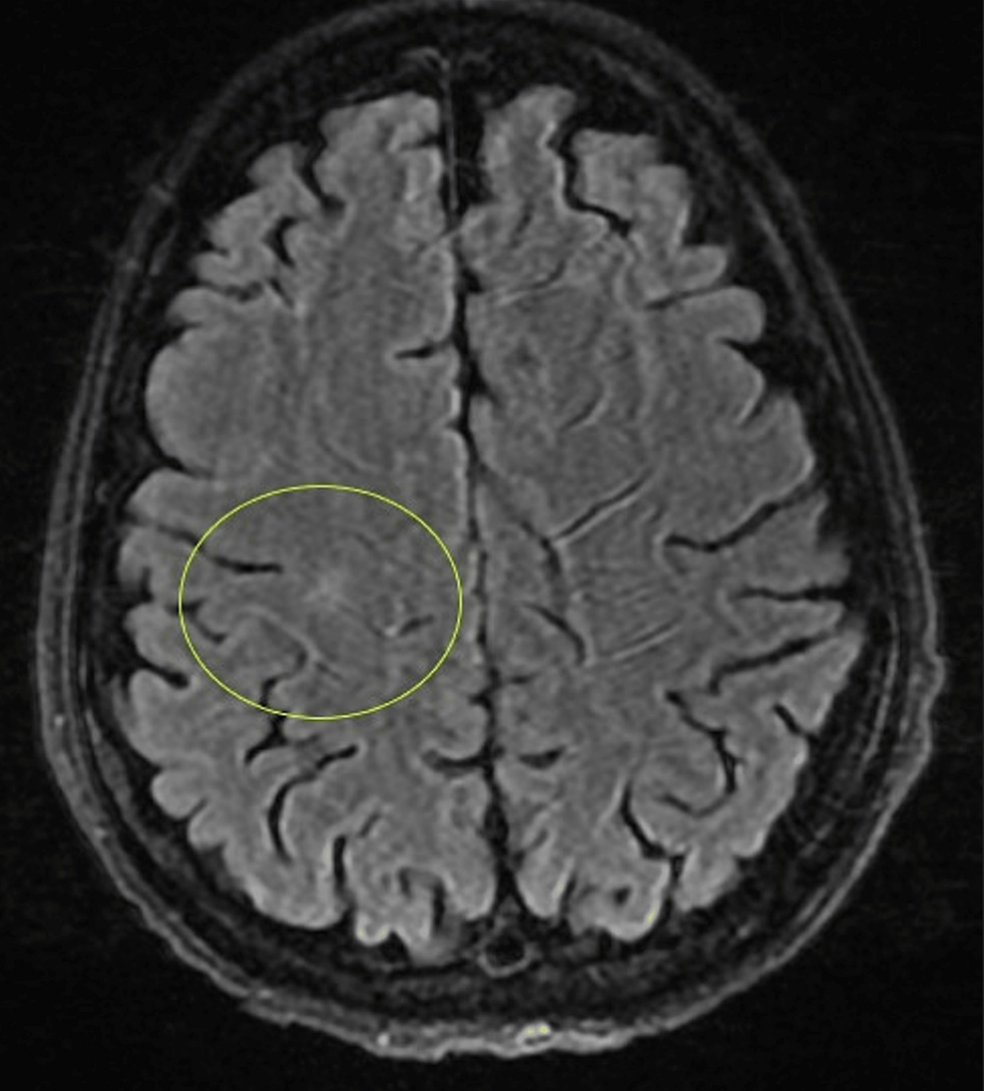
Septic brain embolus.

**Figure 4:**
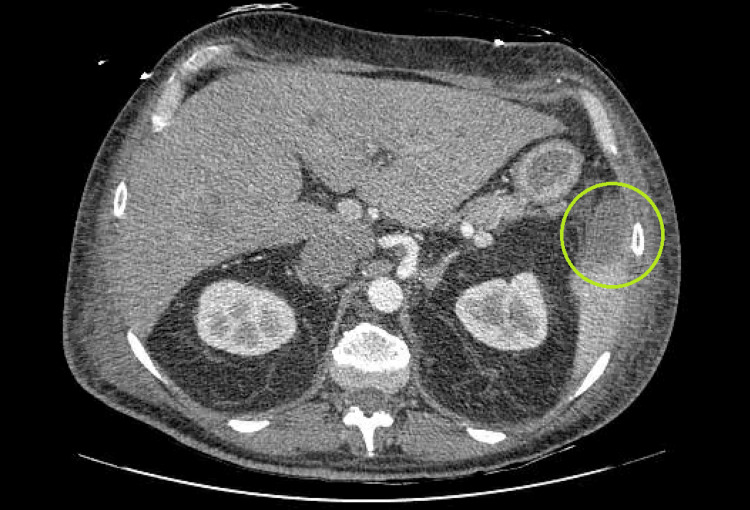
Splenic infarct secondary to septic emboli.

A sepsis workup was initiated for the patient due to elevated white count, tachycardia, and tachypnea which showed *S. epidermidis* bacteremia, and the patient was started on cefazolin. A transesophageal echocardiogram (TEE) revealed aortic and mitral valve vegetations with an anterior mitral leaflet aneurysm and perforation with resultant posteriorly directed severe mitral regurgitation (Figure 2). Further imaging of the brain (Figure 3) and abdomen pelvis revealed septic emboli in the brain and the spleen (Figure 4).

The patient underwent a mechanical aortic and mitral valve replacement. The patient was brought to the intensive care unit on multiple vasopressors but was extubated and weaned off vasopressors on a postoperative day one. The patient was started on oral warfarin for anticoagulation after bridging from a continuous intravenous heparin infusion which was discontinued after the therapeutic international normalized ratio was achieved keeping the patient within the range of 2.5-3.5. The patient was eventually discharged on postoperative day seven with a peripherally inserted central catheter line for antibiotics for a duration of 6 weeks.

The patient returned to the emergency department a few days later for palpitations. He was found to have atrial fibrillation with a rapid ventricular response and was started on intravenous anti-arrhythmic and oral rate-controlling medications. The patient was successfully cardioverted via direct current and was discharged with a Mobile Cardiac Outpatient Telemetry Patch.

## Discussion

Although IE is an uncommon disease, it is known to have high rates of morbidity and mortality and is considered to be one of the most common life-threatening infections [3]. CoNS, which frequently cause prosthetic valve endocarditis, are being recognized more as a cause of NVE [4]. CoNS causing NVE have poor outcomes despite an increase in surgical interventions [5]. Patients with NVE are less commonly caused by CoNS and have an indolent course with fewer embolic events as compared with NVE caused by *Staphylococcus aureus* [5]. One large study with 1500 cases of NVE noted CoNS to be the cause of only 6% of the cases [4].

The Duke’s criteria can be used to diagnose IE which consists of major and minor criteria. Diagnosis of IE can be made when two major, one major and three minor or three minor criteria are satisfied. Major criteria include positive blood cultures with bacteria known to cause endocarditis and echocardiogram evidence of vegetation, abscess, perforation, prosthetic dehiscence, or new valvular regurgitations. Minor criteria include predisposing conditions, fever, Janeway lesions, intracranial hypertension, pulmonary embolism, mycotic aneurysms, Roth spots, Osler nodes, glomerulonephritis, positive rheumatoid factor, positive blood cultures, or positive echocardiogram findings other than those mentioned in the major criteria [6].

A TEE is the preferred modality when compared to a transthoracic echocardiogram (TTE) due to better resolution and visualization of the cardiac structures [7]. Mitral valve perforation is a rare complication of infective endocarditis and the anterior mitral leaflet is the most common structure involved in IE [8]. One of the known complications is leaflet perforation which can occur due to the extension of tissue necrosis [9]. Atrial fibrillation is commonly found in IE which is associated with heart failure. It is also considered to independently cause higher mortality in patients with IE [10].

The brain and spleen are the most frequent sites of embolism in left-sided IE, whereas pulmonary embolism is frequent in right-sided IE of a native valve [11]. Our presented patient had all three thromboembolic events but had evidence for only left-sided endocarditis.

## Conclusions

It was our understanding that our patient had the pulmonary embolism as a result of deep vein thrombosis which was likely provoked in the setting of sepsis and because thrombophilia workup was unremarkable. Additionally, there was no evidence of any shunts that could have caused paradoxical embolus. However, in the setting of septic emboli to the brain and spleen, septic pulmonary embolus could not definitively be excluded. This brings about a unique opportunity to investigate the incidence and probability of left-sided endocarditis resulting in pulmonary emboli.

We also believe it is also essential to closely monitor cardiac function in patients with IE, especially those with a complicated course. Further studies in regards to the use of remote monitoring devices in such cases can help direct medical guidelines which can aid in decreasing mortality related to arrhythmias in this patient population.
